# Mechanisms and Preventative Strategies for Persistent Pain following Knee and Hip Joint Replacement Surgery: A Narrative Review

**DOI:** 10.3390/ijms25094722

**Published:** 2024-04-26

**Authors:** Jasper Murphy, Sery Pak, Lana Shteynman, Ian Winkeler, Zhaosheng Jin, Martin Kaczocha, Sergio D. Bergese

**Affiliations:** 1 Renaissance School of Medicine, Stony Brook University, Stony Brook, NY 11794, USA; jasper.murphy@stonybrookmedicine.edu (J.M.); sery.pak@stonybrookmedicine.edu (S.P.); lana.shteynman@stonybrookmedicine.edu (L.S.); ian.winkeler@stonybrookmedicine.edu (I.W.); 2 Department of Anesthesiology, Stony Brook University, Stony Brook, NY 11794, USA; martin.kaczocha@stonybrookmedicine.edu (M.K.); sergio.bergese@stonybrookmedicine.edu (S.D.B.)

**Keywords:** total knee arthroplasty, total hip arthroplasty, chronic postsurgical pain, central sensitization, chronic inflammation, ketamine, lidocaine, gabapentinoids, SSRIs, local infiltration anesthesia

## Abstract

Chronic postsurgical pain (CPSP) following total knee arthroplasty (TKA) and total hip arthroplasty (THA) is a prevalent complication of joint replacement surgery which has the potential to decrease patient satisfaction, increase financial burden, and lead to long-term disability. The identification of risk factors for CPSP following TKA and THA is challenging but essential for targeted preventative therapy. Recent meta-analyses and individual studies highlight associations between elevated state anxiety, depression scores, preoperative pain, diabetes, sleep disturbances, and various other factors with an increased risk of CPSP, with differences observed in prevalence between TKA and THA. While the etiology of CPSP is not fully understood, several factors such as chronic inflammation and preoperative central sensitization have been identified. Other potential mechanisms include genetic factors (e.g., catechol-O-methyltransferase (COMT) and potassium inwardly rectifying channel subfamily J member 6 (KCNJ6) genes), lipid markers, and psychological risk factors (anxiety and depression). With regards to therapeutics and prevention, multimodal pharmacological analgesia, emphasizing nonopioid analgesics like acetaminophen and non-steroidal anti-inflammatory drugs (NSAIDs), has gained prominence over epidural analgesia. Nerve blocks and local infiltrative anesthesia have shown mixed results in preventing CPSP. Ketamine, an N-methyl-D-aspartate (NMDA)-receptor antagonist, exhibits antihyperalgesic properties, but its efficacy in reducing CPSP is inconclusive. Lidocaine, an amide-type local anesthetic, shows tentative positive effects on CPSP. Selective serotonin reuptake inhibitors (SSRIs) and serotonin norepinephrine reuptake inhibitors (SNRIs) have mixed results, while gabapentinoids, like gabapentin and pregabalin, present hopeful data but require further research, especially in the context of TKA and THA, to justify their use for CPSP prevention.

## 1. Introduction

Arthritis, a leading cause of global disability, highlights the significance of joint replacements, encompassing about 100 conditions affecting joints and surrounding tissues. From 2019 to 2021, 21.2% of U.S. adults (53.2 million) reported diagnosed arthritis, with those aged ≥45 representing 88.3% [1]. Non-surgical interventions, including medication and physical therapy, play an important role in managing arthritis, aiming to improve joint function and reduce pain [2]. Lifestyle changes like exercise and weight management complement medical treatments in addressing arthritis-related challenges [2]. However, despite achieving some success in non-surgical interventions, joint replacement surgery, particularly for the hip and knee, has become a widely performed procedure to alleviate pain, improve function, and enhance the overall quality of life for individuals suffering from severe joint diseases such as osteoarthritis [3].

The increasing prevalence of joint replacement surgeries in recent decades underscores the growing significance of this procedure in the field of orthopedics. Between 2000 and 2019, total hip arthroplasty (THA) increased by 177%, and total knee arthroplasty (TKA) increased by 156% on average [4]. In 2010, the prevalence of total hip and knee replacements in the U.S. stood at 0.83% and 1.52%, respectively. Rates were higher in women, and there was an age-related increase, reaching 5.26% for THA and 10.38% for TKA at eighty years [5]. Additionally, a recent population-based study in the United States concluded that the projected number of total TKAs is anticipated to rise by 143% by the year 2050, even when utilizing a conservative logistic regression model [6]. Osteoarthritis is the primary causative condition for both procedures, while other factors include inflammatory arthritis, fractures, dysplasia, and malignancy [7].

The functional recovery trajectory following joint replacement surgery involves distinct phases with specific challenges and milestones [8]. In the immediate postoperative period, patients typically undergo a process of acute recovery, marked by pain management, wound healing, and initial mobilization. The focus is on early ambulation and rehabilitation to prevent complications such as deep vein thrombosis. Early rehabilitation (weeks to months) intensifies with physical therapy to restore joint mobility and muscle strength, leading to significant pain reduction. The mid-term recovery phase (months) sees continued improvements in strength, stability, and daily activities. Long-term recovery (months to a year) involves refining function and adapting to the prosthetic joint, resulting in near-maximal improvement and reduced pain. Beyond the first year, ongoing maintenance through regular exercise and adherence to medical advice is crucial for sustaining joint health and functional well-being [8].

Despite the significantly improved functional status of most patients undergoing THA and TKA [3,9], chronic postsurgical pain (CPSP) is a common complication of THA and TKA with a recent study finding prevalence to be 20.9% after TKA and 7.5% after THA [10]. The definition of CPSP has evolved over time and was redefined in 2014 to be pain persisting at least three months after surgery, that was not present before surgery, localized to the surgical site or a referred area [11]. CPSP has the potential to induce disability, impair quality of life, generate economic consequences [12], and is consistently identified as a predictor of dissatisfaction following TKA [13]. With the rising prevalence of THA and TKA procedures, the prominence of CPSP has heightened, leading to a focus on current research aimed at understanding its etiology, treatment, and potential preventive measures.

## 2. Risk Factors for CPSP Following TKA and THA

Risk factors for development of CPSP following surgery is a highly studied but incompletely understood area of research. Factors that contribute to the development of chronic pain following TKA and THA may encompass those existing preoperatively, intraoperatively, and postoperatively [14]. Isolating risk factors could lead to targeted preventative therapy and decrease the incidence of CPSP. However, it has been challenging to conclusively identify risk factors and the literature suggests that the etiology of CPSP is multifactorial, involving a diverse array of potential biological, surgical, and psychosocial factors that can impact the outcomes of pain [14].

Ghoshal et al.’s 2023 meta-analysis, comprising 81 studies and 171,354 patients, explored factors influencing CPSP after total knee or hip joint replacement [15]. Results indicated that elevated state anxiety (excluding trait anxiety) 95% CI [0.34, 0.97] and higher Beck Depression Inventory scores correlated with a CPSP following TKA with a 95% CI [0.35, 1.01]. Additionally, a qualitative analysis revealed that heightened preoperative pain scores were linked to CPSP after both TKA and THA [15]. Another study found a robust association between pre-existing diabetes and the development of CPSP after TKA and THA (adjusted OR = 8, 95% CI [2, 38]) [16]. Other studies, however, have not replicated this association with diabetes [15,17,18]. Individual studies of CPSP following THA specifically have identified potential risk factors including intense postoperative pain [19], other pain sites [19], physical functioning at time of operation [20], and preoperative psychological state [20].

The prevalence of CPSP after TKA is higher than that after THA, 0.83% and 1.52%, respectively, as of 2010 [21]. Consequently, more research has been devoted to investigating risk factors specifically associated with TKA. A systematic review and meta-analysis from Lewis et al. found that preoperative pain emerged as the primary predictor associated with persistent pain in both uni- and multivariable analyses (*p* < 0.001) across multiple statistical categorizations [17]. Significant effects were also observed for other pain sites, catastrophizing, mental health, and comorbidities [17]. Another meta-analysis identified sleep disturbances and disorders as a risk factor for CPSP in studies involving TKA and various other surgeries with a small effect size, r = 0.13 (95% CI [0.06, 0.20]) [18]. In addition to the factors above, individual studies have identified more risk factors that may be associated with CPSP following TKA including acute postoperative pain [22,23,24,25], female gender [26], having received at least a high school diploma [23], lower pain threshold [27], pain on walking [23], absence of regular physical activity [23], young age [26], age over 80 [24], BMI above 30 [24], and revision as opposed to primary surgery [28].

## 3. Potential Mechanisms of CPSP

### 3.1. Differential Diagnosis of Pain after Surgery

Although TKA and THA are meant to alleviate pain, they may introduce their own causes of chronic pain that can persist long after surgery if unaddressed. According to a study that followed 2534 knees through primary TKA and a year of recovery, 3% of knees experienced pain due to infection and 12% experienced aseptic pain for at least a year post-surgery. Pain in more than half of the latter group could be attributed to causes within the replaced joint, including prosthesis loosening, polyethylene wear, instability, recurrent hemarthrosis, stiffness, patellar maltracking, and tendon rupture [29]. Similarly, instability, infection, and aseptic loosening represent the top three causes of femoral failure following THA; afflicted patients typically present with pain in the affected joint that worsens with joint loading [30]. Both septic and aseptic causes of pain are addressed through revision surgery, which mitigated the persistent pain of the majority of aseptic TKA patients in the aforementioned study. However, revision TKA is also associated with a higher implant failure rate and risk of complications that could lead to the maintenance or recurrence of chronic joint pain [31,32]. Another possible postoperative complication of TKA is neuromatous pain arising from damage to the surrounding nerves during surgical manipulation—often the infrapatellar branch of the saphenous nerve (IBSN) [33,34]. Damage to this nerve most commonly causes paresthesia but can create a neuroma that results in neuralgia and hyperalgesia in the infrapatellar region [35]. In TKAs accomplished by a standard midline approach, the IBSN is guaranteed to be encountered by the surgeon [36]; a study that followed one surgeon across 127 TKA cases found the incidence of IBSN damage to be 9.7% for primary TKA and 21% for revision TKA [37]. IBSN neuroma is resolved by surgical denervation, which has been shown to drastically reduce patients’ pain [34,35]. Surgical causes of chronic pain after TKA and THA should be ruled out through a combination of a physical exam, serological testing, and radiologic imaging of the implant and surrounding soft tissues prior to further consideration of pain etiology [38].

### 3.2. Central Sensitization

Non-surgical persistent pain following joint replacement may be explained in part by central sensitization (CS). CS is the potentiation of neurons relaying pain signals in the CNS in response to peripheral soft tissue damage and inflammation. CS is characterized by the improper perception of pain in response to innocuous stimuli (allodynia) and a heightened sensitivity to pain stimuli at (primary hyperalgesia) and away from (secondary hyperalgesia) the inciting area of injury [39]. The sustained firing of small-diameter C-fiber nociceptors (as in chronic pain) leads to the temporal summation of postsynaptic potentials in central pain projecting neurons of the dorsal horn of the spinal cord. The resulting depolarization activates N-methyl-D-aspartate (NMDA) receptors by removing the Mg^2+^ block, initiating a Ca^2+^ signaling cascade [40]. Intracellular changes resulting from the cascade are responsible for increasing the sensitivity and receptive field of central pain projecting neurons, contributing to allodynia and secondary hyperalgesia [41].

The leading methods of diagnosing CS are through quantitative sensory testing (QST), the central sensitization inventory (CSI), and/or magnetic resonance imaging (MRI) [42]. It has been consistently demonstrated that CS is associated with higher preoperative pain in patients with osteoarthritis [43].

In a recent meta-analysis that spanned 5 studies and 903 patients, it was demonstrated that patients displaying preoperative CS suffered from more severe pain at 3 months and 1 year post TKA compared to non-CS patients [42]. Furthermore, other studies have shown that patients with CS are less likely to report an improvement in quality of life or satisfaction following TKA [44,45]. In terms of postoperative pain and dissatisfaction, this also rings true for revision TKA [46]. This same phenomenon has been less extensively studied in hip osteoarthritis patients, but evidence suggests a similar association between CS and post-THA pain [43,47]. Recently, Imagama et al. were able to demonstrate a strong association between the degree of preoperative CS and postoperative joint awareness in 263 patients that underwent THA [47].

Interestingly, patients suffering from hip and knee osteoarthritis are more likely to display CS than healthy individuals [45,48]. THA and TKA may serve to decrease the prevalence of CS in osteoarthritis patient populations [45], but this outcome is inconsistent [44].

### 3.3. Chronic Inflammation and Synovitis

Synovitis and chronic inflammation have been implicated as contributing factors to a prolonged pain experience following joint arthroplasty. One exploratory study followed forty-six patients 6 months post TKA [49]. MRI findings were assessed for infrapatellar (Hoffa’s) fat pad synovitis and effusion size. Pressure pain threshold, defined as the minimum force applied to induce pain [50], was also assessed. Patients in the moderate-to-severe postoperative pain group demonstrated a higher grade of postoperative Hoffa synovitis and effusion around the prosthetic joint replacement as compared with patients with none-to-mild pain. Those with moderate-to-severe pain displayed a mean MRI Osteoarthritis Knee Score (MOAKS) of greater than 2 out of 3 for Hoffa synovitis, while those with none-to-mild pain displayed a mean MOAKS below 1 [49]. Decreased pressure pain thresholds in the lower leg, increased temporal summation of pain (defined as increasingly greater pain evoked by repetitive stimuli [51]), and impaired conditioned pain modulation were also displayed when compared with patients with none-to-mild CPSP. Of note, Hoffa synovitis grade was found to be associated with CPSP 6 months after TKA. Specifically, in a regression model of Hoffa synovitis and CPSP, the standardized coefficient was 0.276. These findings indicate that CPSP after TKA may be associated with joint-related inflammation [49].

Although there is growing evidence to indicate that cytokines and inflammatory mediators are associated with osteoarthritic pain and progression [52], the association between postoperative pain and cytokine response remains under examination. One study recruited eighty patients 5 years post TKA and divided groups based on average 24 h knee pain intensity. The serum C-Reactive Protein (CRP) level was measured as an indication of inflammation. The high pain group measured a statistically significant elevation of CRP, accompanied by significantly decreased range of motion in the knee. Specifically, the high pain group demonstrated a CRP level of 4.3 mg/L CI [3.2, 5.5] as opposed to the lower pain group measuring 1.7 mg/L CI [1.2, 2.2] [53].

One study examined the cytokine profile of synovial fluid pre and post TKA in patients who did and did not experience resulting chronic pain. A potential predictor of persistent pain was uncovered; on the second day post surgery, a statistically significant difference was found in the levels of four cytokines—interleukin (IL)-10, IL-1β, vascular endothelial growth factor (VEGF), and IL-12/IL-23p40—in patients who developed persistent pain. Specifically, the level of IL-1β on the first postoperative day in patients with persistent pain was 6.3 times greater than in those with mild pain. It was also found that significantly lower IL-10 levels in the synovial fluid collected pre-arthrotomy had an association with development of postoperative persistent pain [54]. These results indicate that there may be a distinct biological response to surgery that may influence or predict longer term outcomes post arthroplasty.

The prevalence of pain post TKA inspired another study to examine the predictive factors of persistent pain. The serum and synovial fluid of 28 patients was examined at baseline and 2 years post TKA for cytokine markers of inflammation. It was observed that greater preoperative synovial fluid concentrations of tumor necrosis factor (TNF)-α, matrix metalloproteinase (MMP)-13, and IL-6 acted as predictors of less pain improvement 2 years following TKA. Thus, patients with greater levels of preoperative inflammatory markers experienced greater postoperative pain. This was found in a cohort of patients with baseline TNF-α at 2.68 ± 1.24 pg/mL, MMP-13 at 119.29 ± 85.81 pg/mL, and IL-6 at 114.94 ± 72.50 pg/mL. The mean change in the Western Ontario and McMaster Universities Arthritis Index (WOMAC) pain score from baseline to 2 years post TKA was 7.3 within a scale of 0 to 20. It was concluded that patients may have an inflammatory profile that characterizes a susceptibility to ongoing pain after TKA [13].

Cytokines have also been implicated in the development of chronic pain post THA. In a recent study by Chadha et al., 10 patients with well-functioning and painless TKA and THA displayed significantly lower IL-1β and IL-2 levels than patients with painful arthroplasties due to aseptic loosening [55]. A separate genetic study examined the variation in cytokine polymorphisms to determine an individual patient’s susceptibility to periprosthetic osteolysis post THA. It was found that carriers of the pro-inflammatory subtype of an IL-6 allele, IL-6174*G, were 2.5 times more likely to develop severe osteolysis post THA than non-carriers [56], a condition known to be associated with increased pain and limitation of function [55]. Carriers of a subtype of TNF-α, called TNF-238*A, were six times more prone to developing THA failure in the form of osteolysis [56]. This study strongly points to an association between inflammatory factors and CPSP.

### 3.4. Other Biomarkers/Mechanisms

The need to identify biomarkers and biosignatures for chronic pain serves as a pivotal aspect in comprehending the underlying mechanisms of CPSP. Numerous biomarkers for CPSP are under investigation, with chronic pain demonstrating significant heritability, estimated between 25 and 50% for various phenotypes [57]. While research on genomic factors specific to CPSP post TKA is still limited, existing studies focus on differences in DNA, particularly single-nucleotide polymorphisms associated with painful conditions. Current research has studied several signaling molecules and receptors interacting with nociceptive pathways. A study by Thomazeau et al. assessed the associations of post-surgical pain and the catechol-O-methyltransferase (COMT) gene, which plays a critical role in degrading catecholaminergic neurotransmitters. They identified a modest correlation between the A allele of the gene and CPSP six months after surgery [23]. Similarly, Bruehl et al. discovered that variations in the potassium inwardly rectifying channel subfamily J member 6 (KCNJ6) gene, which affects G-protein-coupled inwardly rectifying potassium (GIRK) channels that determine degree of analgesia experienced upon opioid receptor activation, correlate with increased opioid use post TKA [58]. However, the pathways of KCNJ6 gene variations on pain phenotypes still require further study.

Lipids, crucial for cellular function, signaling, and energy storage, are increasingly recognized for their role in nociceptive processing and pain resolution [57]. Early research has begun exploring their potential as markers for the acute to chronic pain transition. Costello et al. studied presurgical metabolite ratios related to inflammation, noting distinct differences between pain responders and non-responders. Acetylcarnitine to phosphatidylcholine acyl-alkyl was five times higher in pain non-responders whereas phosphatidylcholine diacyl to isoleucine was twenty-one times lower in pain non-responders than responders, indicating these lipids to be a potential biomarker for predicting chronic pain outcomes [59].

### 3.5. Psychological Aspects of Persistent Postoperative Pain

CPSP imposes substantial physical and psychological challenges on individuals initially seeking improvement or postoperative recovery. In recent years, there has been a consistent identification of various presurgical psychological risk factors associated with CPSP, including anxiety, depression, pain catastrophizing, and general psychological distress. In addition, poor social support has shown to be linked to an increased likelihood of transitioning to chronic pain [57].

Multiple studies have demonstrated that patients with depression, anxiety, somatization, and control beliefs experienced poorer postoperative outcomes [17,60,61,62,63]. A systematic review conducted by Hinrichs-Rocker et al. identified 50 publications that revealed psychosocial predictors that correlated with CPSP. Each study underwent evidence assessment, and corresponding score points were assigned for comparison. Depression, psychological vulnerability, stress, and late return to work exhibited a likely correlation with CPSP [64]. Furthermore, a meta-analysis by Lewis et al. reviewed 32 studies, involving almost 30,000 patients who underwent TKA. Preoperative pain demonstrated a significant effect size >0.1; however, the most substantial effect sizes were observed for catastrophizing (Fisher’s Z = 0.302) and depression (Fisher’s Z = 0.217) [17]. A systematic review by Guisti et al. showed similar results in which their narrative synthesis demonstrated that state anxiety and psychological distress were consistently linked to CPSP. In their meta-analysis, state anxiety (r = 0.24), trait anxiety (r = 0.13), mental health (r = −0.17), depression (r = 0.16), and catastrophizing (r = 0.19) all had statistically significant associations with CPSP with *p* values < 0.001 [65].

Despite the psychological risk factors for CPSP, the current literature does not advocate for routine psychological interventions in TKA and THA. However, the literature is still in its nascent stage. Several studies have been conducted to determine the effectiveness of interventions using pain coping skills. A study by Riddle et al. investigated whether pain coping skills training in individuals with moderate-to-high pain catastrophizing undergoing knee arthroplasty yields improved outcomes 12 months postoperatively compared to standard care or arthritis education [66]. In this randomized control trial, adults with pain catastrophizing undergoing TKA training in cognitive behaviorally based pain coping skills did not yield additional benefits in terms of pain reduction or functional improvement, beyond the substantial improvements achieved with standard surgical and postoperative care [66]. Another study by Wong et al. explored the efficacy of a pain management educational intervention in addressing pain, anxiety, and self-efficacy levels among patients with musculoskeletal trauma and subsequent orthopedic surgery. The experimental group exhibited statistically significant reductions in pain, lowered anxiety levels, and increased self-efficacy during the hospitalization period [67]. Evidently, continuous research efforts aim to determine the extent to which psychological interventions have a positive influence on chronic pain following surgery.

## 4. Potential Preventative Options

Several treatments for the prevention of CPSP have been explored. Evidence on perioperative therapy for prevention of CPSP post TKA/THA is summarized in Table 1.

### 4.1. Multimodal Pharmacological Analgesia

Acute postoperative pain has been associated with the development of CPSP in TKA, THA, and a broad range of other surgeries [68,69,70]. Recognizing this association, researchers have explored effective perioperative and postoperative pain management as a potential preventive strategy. Although multimodal analgesia is currently one of the primary methods for managing postoperative pain following these procedures [71], the incidence of CPSP after utilizing this therapeutic intervention has not yet been thoroughly examined clinically and necessitates additional study. This method utilizes predominantly nonopioid analgesics, aiming to achieve an additive or synergistic effect that enhances pain relief while concurrently diminishing opioid utilization and associated side effects [71,72]. The standard multimodal regimen begins in the preoperative phase, with the administration of a combination of acetaminophen, non-steroidal anti-inflammatory drugs (NSAIDs), and a gabapentinoid [72]. In the postoperative stage, the regional analgesic technique is continued, along with acetaminophen, an NSAID, and a gabapentinoid [72]. Moreover, the administration of intraoperative steroids has emerged as a promising adjunctive approach to minimize postoperative pain. The use of intraoperative steroids has shown substantial acceleration of rehabilitation, knee strength, and a concurrent reduction in opioid requirements [73]. This nuanced approach, in contrast to the historical preference for epidural analgesia, has garnered prominence due to documented limitations associated with the latter in orthopedic surgeries, such as TKA. Studies indicate that epidural analgesia is linked to prolonged postoperative recovery and a consequential “motor blockage” presenting as knee buckling, impairing subsequent ambulation [71].

The shift towards multimodal analgesia, specifically tailored for postoperative pain management after TKA, is substantiated by multiple studies. The emphasis on NSAID and acetaminophen within the multimodal approach has demonstrated tangible benefits, including reductions in perioperative opioid use for THA and TKA [74,75]. A pivotal component of multimodal analgesia, preemptive analgesia, is also a notable contributor to enhanced outcomes. Studies conducted by Xu, Li, and Zheng indicate that preemptive analgesia not only results in improved joint function and pain control but also facilitates a faster postoperative functional recovery, underscoring its integral role within the multimodal approach [76]. Perioperative local infiltrative analgesia (LIA) has also been investigated with less promising results. Out of six LIA randomized controlled trials in either TKA or THA that were identified through the literature review, five demonstrated no favorable reduction in long term postoperative pain [77,78,79,80,81]. One study which examined both TKA and THA found that the LIA group in the hip trial had significantly less pain at 12 months compared to standard care while there was no significant difference between groups in the knee trial [82].

### 4.2. Regional Anesthesia

Administering local anesthetics near nerves, nerve bundles, or roots blocks sodium channels in peripheral nerves, interrupting pain signal transmission from the surgical site to the central nervous system. This may help prevent CPSP by effectively managing acute pain [83]. Unfortunately, local postoperative pain control has produced mixed results with regards to the incidence of chronic pain and has not been shown to be directly effective. According to a recent systematic review, out of 11 nerve block studies following TKR, only 1 showed evidence favoring femoral nerve block (FNB) for CPSP at 6 months and this effect was not sustained at 1 year [84]. The successful trial included 280 patients with half receiving a continuous femoral nerve analgesia of 5 mL of 0.15% ropivacaine followed by an infusion of 0.15% ropivacaine at 5 mL/h, with a bolus of 5 mL and lock time of 30 min [85]. In comparison to the control group of intravenous patient-controlled analgesia, the continuous FNB showed a decreased incidence of CPSP from 38.5% to 25.7% at 6 months (*p* = 0.021) but not 1 year (*p* = 0.273) [85]. Of the other studies showing no significant results, tested blocks included the FNB continuous, FNB single, adductor canal block, and sciatic nerve block [84]. While the rates are low, potential adverse effects of nerve blocks include hematoma, infection, peripheral nerve injury, and systemic toxicity [86]. Additionally, there is some concern that motor blockade as a result of regional anesthesia may delay postoperative ambulation and indirectly contribute to persistent pain following TKA and THA [87], especially with the increasing implementation of same-day physical therapy [88]. However, this matter has not been extensively investigated.

### 4.3. Ketamine

Ketamine is an NMDA-receptor antagonist that is frequently employed as a dissociative anesthetic and has been more recently utilized for treatment-resistant depression and other psychiatric conditions [89,90]. Ketamine is proposed to reduce CPSP through its antihyperalgesic and anti-central sensitization properties, which are attributed to the inhibition of the upregulated NMDA receptor in chronic pain states [91]. Ketamine blocks NMDA receptors through two mechanisms: it reduces the mean open time of the channel by blocking the open channel, and it decreases the frequency of channel opening by binding to the closed receptor through an allosteric mechanism [92]. Antihyperalgesic agents work by modulating the nervous system’s processing of pain signals, ultimately diminishing the perception of pain or preventing the development of heightened sensitivity. Through this mechanism, ketamine suppresses hyperalgesia and central sensitization to pain following surgery [93]. However, a reduction in CPSP has not been conclusively supported by research.

Usage of perioperative ketamine to decrease CPSP has demonstrated mixed results in TKA, THA, and mixed surgical studies. In a randomized controlled trial comprising 70 patients in the placebo group and 72 in the ketamine group (IV ketamine before incision 0.5 mg/kg and a 24 h infusion 2 microg/kg/min), a significant reduction in CPSP was observed six months after THA [94]. On day 180, rest pain in the operated hip was experienced by 21% of patients in the placebo group compared to 8% in the ketamine group. Another trial randomized patients to receive either ketamine or placebo in association with spinal anesthesia for TKA and found a decrease in pain in the ketamine group at 6 months [95]. Notably, this was a small pilot study of only twelve total patients. On the other hand, another RCT randomized 75 TKA patients who received either a 48 h infusion of ketamine, nefopam, or placebo. The follow-up study assessed patients at 6 and 12 months and found that the prevalence of chronic pain at 12 months was 17.4%, with no significant difference between the ketamine (12.5%), nefopam (13.7%), and placebo groups (26.1%) [96]. Further large randomized controlled trials and meta-analyses are necessary to determine the efficacy of ketamine for CPSP specifically for THA and TKA.

### 4.4. Lidocaine

Lidocaine is a local anesthetic and antiarrhythmic medication. It belongs to the class of amide-type local anesthetics and is commonly used to induce local anesthesia. Lidocaine works by inhibiting the voltage-gated sodium channels, preventing the initiation and conduction of nerve impulses [97]. While there is very little research specific to TKA and THA, perioperative lidocaine has shown tentatively positive effects on CPSP following other surgeries. In a network meta-analysis of non-opioid analgesics, intravenous lidocaine (12 RCTs) showed the largest significant difference from the placebo in preventing CPSP at ≤6 months (OR = 0.32; 95% credible interval CrI [0.17, 0.58]). The included lidocaine studies used dosages of 1.5mg/kg STAT followed by intraoperative dosing ranging from 1.5 mg/kg/h to 3 mg/kg/h. One study followed intraoperative dosing with 1 mg/kg/h for 24 h. However, confidence in these findings is limited due to issues with the risk of bias and imprecision, and there were minimal data for longer-term reductions in pain [98]. In two additional meta-analyses examining perioperative intravenous lidocaine infusion, a reduction in CPSP at greater than 3 months was observed in the lidocaine group following breast surgery (four RCTs) [99] and general surgery (six RCTs) [100]. More analysis is needed to quantify the benefit of perioperative lidocaine and justify its role in clinical practice, especially in THA and TKA specifically.

### 4.5. SSRIs and SNRIs

The prospective analgesic mechanism of selective serotonin reuptake inhibitors (SSRIs) and SNRIs in preventing CPSP involves increasing spinal monoamine concentration, allowing serotonin and norepinephrine to act on receptors in the spinal cord to reduce the intensity of pain signals [101]. Specifically, serotonin and norepinephrine act on 5-HT and adrenergic receptors causing the inhibition of adenylyl cyclase, opening of potassium channels, closing of calcium channels, and modulation of neurotransmitter release, leading to a decrease in neuronal excitability [101]. By modulating pain pathways, increased monoamine concentration helps to counteract hyperalgesia and prevent central sensitization in the aftermath of nerve injuries, thereby normalizing the response to pain stimuli [102].

Trials of perioperative SSRIs and SNRIs for CPSP reduction show mixed results. Limited research has been conducted to explore the efficacy of these agents in the context of THA and TKA. Two randomized controlled trials of the SNRI duloxetine demonstrated no significant differences in chronic pain following THA or TKA [103,104]. One trial used 60 mg/day for 10 weeks preoperatively [103] while the other used 60 mg orally daily for 15 days postoperatively [104]. Additionally, 21% of the Reinstra et al. intervention cohort discontinued duloxetine due to adverse effects [103]. SSRIs and SNRIs have shown more success in the context of surgery as a whole, with three separate meta-analyses including a variety of dosing regimens concluding that there was a significant reduction in CPSP following perioperative administration of either SSRIs or SNRIs [102,105,106]. Additional research focused specifically on TKA and THA is needed to determine the generalizability of the results from these meta-analyses to joint replacement surgery.

### 4.6. Gabapentinoids

Gabapentinoids, including gabapentin and pregabalin, have been investigated to a certain extent for their potential role in preventing CPSP. Gabapentinoids are posited to mitigate chronic pain by modulating synaptic transmission and decreasing the release of excitatory neurotransmitters, which attenuates the neuronal hyperexcitability associated with CPSP [107]. Specifically, gabapentinoids bind to the α2-δ subunit of voltage-gated calcium channels in the central terminals of primary sensory neurons that project to the dorsal horn. This interaction inhibits the influx of calcium ions into presynaptic neurons, thereby reducing neurotransmitter release [108].

Two randomized controlled trials of perioperative gabapentinoids used in TKA were identified through the literature review. In a study involving 240 patients, a statistically significant reduction in the incidence of CPSP by 5.2% at 6 months [109] was observed with a regimen of oral pregabalin (300 mg) administered before TKA and continued for 14 days after TKA (50-150 mg twice daily). The other study of 60 patients found no significant effect on CPSP with an almost identical regimen [110]. A meta-analysis including eight studies of the use of perioperative gabapentinoids in surgery as a whole presented hopeful data showing a moderate-to-large reduction in CPSP [111]. Six gabapentin trials demonstrated a substantial decrease in the occurrence of CPSP, with a pooled OR of 0.52 (95% confidence interval CI [0.27, 0.98]; *p* = 0.04). Two pregabalin trials also indicated a large reduction, with a pooled OR of 0.09 (95% CI [0.02, 0.79]; *p* = 0.007). Variability in dosing regimens was observed given the various different surgeries, with some studies employing a single preoperative dose and others maintaining daily dosing for a duration of up to 2 weeks postoperatively [111]. While the data on perioperative gabapentinoids are tentatively hopeful, more research is necessary, especially in the context of THA and TKA, to justify the use of gabapentinoids for the prevention of CPSP.

**Table 1:** Current evidence on perioperative therapy for prevention of CPSP post TKA/THA.

Intervention	Support for Prevention	Summary of Evidence
Multimodal pharmacological analgesia	Theoretical (unexplored in THA/TKA)	Postoperative pain is associated with CPSP following TKA/THA [19,22,23,24,25]. Multimodal analgesia is a leading strategy for reduction of postoperative pain [68,71,72,73,74,75].
Regional Anesthesia	Mixed, largely negative	Of 11 nerve block and LIA studies identified, 1 showed favorable evidence. Ten showed no difference [84].
Ketamine	Mixed	Two RCTs showed reduction in CPSP at 6 months following perioperative ketamine [94,95]. One RCT showed no difference at 6 or 12 months [96].
Lidocaine	Positive in other surgeries, unexplored following THA/TKA	One meta-analysis w/12 RCTs found reduction in CPSP at <6 months. Two meta-analyses (6 RCTs and 4 RCTs) saw reduction at >3 months.
SSRIs and SNRIs	Negative in THA/TKA, positive in other surgeries	Two RCTs w/negative results following THA/TKA [103,104]. Three meta-analyses in other surgeries w/positive results [102,105,106].
Gabapentinoids	Mixed	One RCT of 240 patients found reduction in CPSP at 6 months [109]. Another RCT of 60 patients found no significant effect [110].

## 5. Therapeutic Options

There are various pharmacological and interventional approaches for treating CPSP, drawing upon established treatment options for other chronic pain conditions [112]. Pharmacological treatment typically begins with a non-opioid analgesic that evolves toward more potent analgesic medications depending on the patient’s response [112]. Given the neuropathic etiology frequently associated with CPSP, therapeutic strategies predominantly target neuropathic pain pathways. Primary pharmacological agents recommended include tricyclic antidepressants, serotonin-norepinephrine reuptake inhibitors (SNRIs), and gabapentinoids, with tramadol and tapentadol reserved for secondary therapy [112,113]. Interventional methods have been increasingly studied for their potential effectiveness. Peripheral nerve stimulation (PNS) at the saphenous nerve branches has emerged as a straightforward, selective, and safe technique for patients with chronic knee pain [114]. In a limited study conducted by Früh et al., participants who underwent PNS at the saphenous nerve branches had reduced opioid usage and significant improvements in quality of life, mood, and sleep [114]. Additionally, neurolysis of the genicular nerve using radiofrequency is a potential treatment that has shown positive initial results in reducing pain and improving joint function [115]. However, the widespread use of these interventional strategies is limited by the need for more thorough research.

## 6. Conclusions

CPSP remains a pervasive challenge across various surgical procedures, extending beyond THA and TKA. Its manifestation varies, encompassing mild irritations following an otherwise successful surgery to the more severe and potentially debilitating state of chronic pain. Despite extensive research into CPSP, the findings have been inconclusive in terms of identifying definitive risk factors, understanding its etiology, establishing effective preventative measures, and determining optimal therapeutic options. The complexities surrounding CPSP necessitate a continued commitment to research. By refining our understanding of the underlying mechanisms and risk factors, researchers and clinicians can work collaboratively to formulate more effective preventative strategies and therapeutic interventions. This ongoing dedication to research is crucial in the collective effort to reduce the incidence of CPSP, ultimately enhancing the postoperative experience for patients undergoing various surgical procedures.

## Data Availability

No new data were created or analyzed in this study. Data sharing is not applicable to this article.
